# Private by default: reasonable expectations in secondary uses of patient data

**DOI:** 10.1093/medlaw/fwaf038

**Published:** 2025-10-31

**Authors:** Miranda Mourby

**Affiliations:** Centre for Health, Law & Emerging Technologies, Faculty of Law, University of Oxford, Oxford, OX1 3TJ, United Kingdom; School of Law, University of Sheffield, Sheffield, United Kingdom

**Keywords:** confidentiality, health data, human rights, misuse of private information, privacy, reasonable expectations

## Abstract

The ‘reasonable expectations of privacy’ test has become central to English information law. The fact-specificity of this test has obfuscated the scope of patients’ privacy rights. In both *R (W, X, Y & Z) v Secretary of State for Health* and *Prismall v Google*, the claimants were found to lack a circumstantially reasonable expectation of privacy when their identifiable information was disclosed outside the healthcare system, obviating the need for justification under Article 8 European Convention on Human Rights (ECHR). In response to these developments, this article argues for a legal presumption of privacy when patients’ data are used for purposes other than their healthcare. This would be a development of the courts’ existing ‘starting point’ of assuming reasonable expectations of privacy in identifiable medical information. The two cases explored in this article suggest that this ‘starting point’ is not enough, and still affords judges broad discretion to evaluate a (non-exhaustive) list of factors in each individual case. For the sake of the clarity and accessibility of patients’ rights, I argue that privacy should be presumed by default when their data are used for purposes other than their healthcare.

## I. INTRODUCTION

In 2015, Lord Kerr warned that making ‘reasonable expectations of privacy’ an inflexible, wholly determinative test for the application of privacy rights risked unwarrantably prescribing their scope.^1^ Recent English case law seems to have borne out this concern, particularly as regards data in the National Health Service (‘NHS’), leaving patients’ privacy at the mercy of how judges might construe their ‘reasonableness’.

As a response, this article advocates for a legal presumption of privacy when patients’ data are used for purposes other than providing care. This would be consistent with existing judicial comments (eg in *Prismall v Google*) that a reasonable expectation of privacy should be the ‘starting point’ in such cases.^2^ But a presumption would be stronger than this ‘starting point’: it would place the onus on a party claiming an absence of rights, to show that patients’ privacy is not impacted by the use of their information.

This enhanced protection would not only be doctrinally consistent with the purpose of Article 8 European Convention on Human Rights (‘ECHR’), it is also necessary given the expansion in secondary uses of health data. ‘Secondary’ uses of health data encompasses all purposes other than providing healthcare (the ‘primary’ use of information contained in health records).^3^ These secondary uses of health data have a significant role to play in the promised development of Artifical Intelligence (‘AI’)-assisted medicine, hence why the EU has introduced a bespoke regulatory framework.^4^ Given this acknowledged need for regulation of further uses of patients’ records, it is concerning that English case law seems to be contracting into technicalities around scope. I suggest the core reason for this contraction is the crystallization of ‘reasonable expectations’ from an indicative factor for the application of privacy rights into a rigid, definitive test.

This criticism is presented primarily in the context of two cases, in which the disclosure of significant volumes of identifiable patient information for secondary purposes was deemed to fall outside the scope of the right to private life under Article 8 of the ECHR. The first of these cases is *R (W, X, Y & Z) v Secretary of State for Health*.^5^ In this 2015 judgment, the Court of Appeal ruled that the claimants had no reasonable expectation of privacy vis-à-vis the Home Office, meaning their information was not considered confidential when it passed from the NHS to the Secretary of State. This was based on the logic of the claimants’ reasonable anticipation of the outcome, despite the potential impact on their immigration status, and thus their ability to see their families. This focus on rational anticipation neglects, in my view, the central purpose of Article 8: the protection of private and family life.

The ‘reasonable expectations’ test is also used for the ‘misuse of private information’, or ‘MOPI’, tort, and was applied in the High Court judgment in *Andrew Prismall v Google UK Ltd*,^6^ where a claim brought on behalf of 1.6 million patients was struck out because the hypothetical ‘lowest common denominator’ patient was found to lack a reasonable expectation of privacy. The construction of ‘reasonableness’ is considered here, with its lineage in English law going back to the Edwardian ‘reasonable man’ casting a long shadow on judicial construction of the ‘ordinary, reasonable’ perspective. This judgment has since been upheld by the Court of Appeal, albeit with a general comment about privacy in medical records cited above:

The starting point is that there will normally be a reasonable expectation of privacy for any patient identifiable information in medical notes*.^7^*

This ‘starting point’ should be formalized as a legal presumption within confidentiality and MOPI claims. As I will explain in Section II, elevation to a ‘presumption’ would stabilize this starting point, by requiring at least some evidence to displace it. Otherwise, we risk continuation of the trend suggested by *R (W, X, Y & Z)* and *Prismall*, where large-scale disclosures of patient data outside the NHS did not require public interest justification under Article 8(2) ECHR, because patients’ rights were deemed inapplicable.^8^ If these rights are not brought back into alignment with the purpose of Article 8—to protect private life—they will remain vulnerable to summary dismissal based on a technical, non-purposive construction of their scope.

The next section provides an overview of reasonable expectations in English privacy law, and explains further why I suggest a legal presumption is required for patients’ identifiable data. Sections III and IV will then (respectively) consider the judgments in *R (W, X, Y & Z)* and *Prismall v Google*, which both demonstrate the consequences of disapplying Article 8 on a highly discretionary construction of ‘reasonable expectations’.

## II. REASONABLE EXPECTATIONS OF PRIVACY

In 2004, the (then) UK House of Lords famously confirmed in *Campbell v MGN* that:

the touchstone of private life is whether **in respect of the disclosed facts** the person in question had a reasonable expectation of privacy^9^ (emphasis added)

In the judgments that have followed, this fact-sensitive ‘touchstone’ of reasonable expectations of privacy has been applied on a case-by-case basis, and become an essential precondition for the application of Article 8 in English case law. It has shaped the subsequent 20 years of judgments on Article 8 ECHR, culminating in the (now) UK Supreme Court’s confirmation in *Bloomberg v ZXC* that demonstrating a circumstantially reasonable expectation of privacy is the initial stage of the test of for a successful claim under the MOPI tort.^10^

### A. Fact-specific expectations

The fact-specific nature of the English approach to Article 8(1) ultimately renders the category of ‘private information’ indeterminate. The Supreme Court has confirmed that there are no exhaustive factors which can be taken into account to ascertain a reasonable expectation of privacy, and the weight to be attributed to each factor will vary in each case.^11^ This puts potential claimants in a difficult position. As will be explored throughout this article, while any *interference* with Article 8 must be accessible and foreseeable (if it is held to be engaged),^12^ it is difficult to foresee with any degree of certainty when the courts will deem Article 8 engaged in the first place. This makes the scope of the right far less predictable and accessible, making it hard to say for certain when secondary uses of data will require legal justification under confidentiality and MOPI law.

This expectations-based approach is not inevitable, however. As explored further in Section IV, there have been multiple cases in which the European Court of Human Rights (‘ECtHR’) has found that Article 8 applied to a set of facts without any reference to reasonable expectations of privacy.^13^ In some, there was no weighing of factors at all: the mere finding that physical or moral integrity had been impacted was sufficient.^14^ In multiple judgments, the ECtHR has stated that reasonable expectations of privacy are a significant but not conclusive factor in the application of Article 8 ECHR.^15^ On the author’s review of the Strasbourg case law, it appears that many of the references to ‘reasonable expectations of privacy’ in ECtHR judgments come from litigation against the UK, in which the legal submissions were inevitably framed in these terms because of the domestic emphasis on our ‘touchstone’ for Article 8.^16^

The divergence between the UK and the ECtHR on the significance of reasonable expectations of privacy under Article 8 was acknowledged by Lord Kerr in 2015,^17^ but this has not prevented its consolidation into the MOPI tort in *Bloomberg.* There is one way of reconciling these two approaches, however: recognition of the courts’ ‘starting points’ in constructing reasonable expectations as more formal legal presumptions that Article 8 will apply in certain circumstances. In essence, this would mean that ‘reasonable expectations of privacy’ in identifiable patient information need only become a determinative issue when a defendant has some evidence to contest their default application.

Recognition of a legal presumption of reasonable expectations—at least in prescribed circumstances—is a less radical transition than it may appear from the ‘headline’ touchstone in *Campbell.* The ostensibly nebulous and fact-specific approach to reasonable expectations of privacy has already been (relatively) solidified by the development of what the courts have variously called ‘starting points’,^18^ ‘general rules’,^19^ ‘principles’,^20^ and ‘predispositions’^21^ in the construction of reasonable expectations of privacy. The argument of this section is essentially that these would be better acknowledged as legal presumptions that Article 8 will apply in certain scenarios (which are likely to recur).

One such commonly recurring scenario would be the use of patients’ identifiable information for purposes beyond their healthcare. Hence the overarching argument of this article: that for combined purposes of policy, clarity, and accessibility, there should be an acknowledged legal presumption of reasonable expectations of privacy in secondary uses of identifiable patient data.

### B. ‘Starting Points’ and legal presumptions

The word ‘presumption’ is used in varying ways across legal jurisdictions, but a minimal account of the term would be:

a legal mechanism which, unless sufficient evidence is introduced to render the presumption inoperative, deems one fact to be true when the truth of another fact has been established.^22^

This is the bare bones of the term ‘presumption’, as it is used in this article. Within English common law, there are more precise categories of legal presumption. For the present purposes, the most relevant type would be an ‘evidential presumption’, under which a conclusion must be drawn in the absence of any evidence to the contrary.^23^ It does not completely reverse the burden of proof, but once the party relying upon it has demonstrated a basic fact (eg that the information in question was derived from an identifiable patient record), the onus shifts to the opposing party to adduce counter-evidence against the presumption^24^ (ie that the information should nonetheless fall outside the scope of Article 8).

In practical terms, this would mean that information demonstrably derived from a patient’s identifiable medical record would be presumed to attract a reasonable expectation of privacy, unless evidence was adduced to the contrary (for example, because the opposing party can show that the patient had made the information public, and it should no longer be reasonably considered private^25^). A defendant would have the choice to contest the reasonable expectation of privacy under Article 8(1), and present some evidence to do so, or simply justify their decision under Article 8(2). The policy benefit of clarifying this choice is that—by implication—custodians of patients’ data will have reduced scope to imagine that there may be specific facts that will disapply patients’ privacy rights, and should instead presume by default that secondary uses of identifiable data will require justification as necessary and proportionate.^26^ As explored further in Section IV, this wards against disproportionate disclosure of patients’ identifiable information.

### C. Bloomberg and presumptions

The main problem with the argument outlined above is that the Supreme Court has, in *Bloomberg*, explicitly stated that their ‘legitimate starting point’ or ‘general rule’ regarding a reasonable expectation of privacy was not a legal rule or a legal presumption.^27^ This litigation concerned media reporting during a criminal investigation. It represents broad, important authority on the MOPI tort, which counts against a legal presumption of reasonable expectations for any kind of data. There are, however, two reasons for distinguishing the Supreme Court’s statement, and nonetheless presenting this argument here.

Firstly, the Supreme Court judgment in *Bloomberg* was about reasonable expectations of privacy in the context of criminal investigations, and so their rejection of a legal presumption was technically only of a presumption in these circumstances (albeit with implications for other contexts). The specific policy grounds for a presumption of privacy in identifiable patient data would not have been considered, including the importance of preserving medical confidentiality,^28^ and the fact that information concerning health are generally viewed as a core aspect of private life.^29^ Adding to these oft-observed grounds for the application of Article 8 ECHR to medical data, this article will also explore the consequences of *not* automatically applying Article 8 in *R (W, X, Y & Z)* and *Prismall v Google*, and the gaps in legal protection this has created.

More broadly, however, there is also a plausible argument that the Supreme Court *did* in fact apply a presumption in *Bloomberg*, but declined to use this label to preserve the alleged fact-specificity of the ‘reasonable expectations’ test. In considering whether the Bloomberg media company had (to paraphrase their words) ‘successfully challenged the legitimate starting point’ of reasonable expectations of privacy, the Supreme Court began:

In summary Bloomberg challenges the general rule or legitimate starting point in relation to this category of information on the following bases^30^

This language is very close to that of a legal presumption. The main difference, for our present purposes, was that the ‘bases’ submitted by Bloomberg were legal submissions on reasonable expectations, and not factual evidence. Otherwise, the Court appears to be setting a model under which a ‘general rule or legitimate starting point’ has to be ‘challenged’, but the success of this challenge is deliberately not framed as a rebuttal. There is a striking tentativeness in the phrasing here. The Court did not choose between ‘legitimate starting point’ or ‘general rule’ in their judgment, almost in acknowledgement that neither phrase is a perfect description for the legal mechanism at play.

Ultimately, it is beyond the scope of this article to say whether the Supreme Court should have called their ‘legitimate starting point or general rule’ a legal presumption, at least in the context of information relating to criminal investigations. The argument presented here makes a policy case for a presumption to preserve medical confidentiality, acknowledge the default importance of this information for private and family life, and align UK privacy law with the ECtHR.

There may well be arguments to be made for presumed reasonable expectations of privacy in other contexts. For example, the courts have come very close to establishing a presumptive ‘starting point’ of reasonable expectations of privacy for children who are photographed without parental consent. This comes originally from *Murray v Express Newspapers*, where the Court held that:

subject to the facts of the particular case, the law should indeed protect children from intrusive media attention, at any rate to the extent of holding that a child has a reasonable expectation that he or she will not be targeted in order to obtain photographs^31^

Although couched in fact-specific language, there was still a recognized principle of how the law ‘should’ protect children by attributing a default^32^ reasonable expectation of privacy in such circumstances. Similarly, in *Weller*^33^ it was established that ‘*without more*’, the celebrity status of a child’s parents should not be taken into account in determining their reasonable expectations of privacy. These starting points are clearly driven by a recognition of the core interests in private life that Article 8 ECHR seeks to protect. Formalization of these starting points into legal presumptions for children could likewise stabilise the boundaries of ‘private information’, at least within commonly recurring circumstances where Article 8 ECHR should be foreseeably recognized as engaged.

If followed to the letter, the fact-specific test set by the senior courts in *Campbell* and *Bloomberg* could make the scope of Article 8 entirely contingent on a discretionary valuation of non-exhaustive factors. Without the clear, explicit legal mechanism of a presumption, there is little guarantee that the values within Article 8 ECHR will, in fact, stabilize its application in scenarios where people’s private and family lives are significantly impacted as a matter of routine. Given that Article 8 underpins both common law confidentiality and the MOPI tort, this lack of clarity currently has implications for both areas of law. This is explained briefly in the next subsection.

### D. Reasonable expectations in medical confidentiality

It is generally taken as axiomatic that information disclosed by patients to their doctors attracts a duty of confidence.^34^ It is less clear, however, what this quality of ‘confidentiality’^35^ means in English law. Even following the key authoritative guidance on the topic, the medical duty of confidence could be characterized as ethical,^36^ contractual,^37^ tortious,^38^ equitable,^39^ or statutory^40^ in origin and nature. It can also be seen as a manifestation of the right to private and family life under Article 8 European Convention on Human Rights.^41^ The heterogeneity of the law and ethics surrounding confidentiality has real implications for the clarity of the legal doctrine.

For example, following the NHS Royal Free’s disclosure of patient data to Google DeepMind (explored in Section IV of this article, as the background to the *Prismall* litigation), Linklaters LLP prepared a report which suggested that clinicians could be subject to both an equitable duty of confidence, and a tortious duty not to misuse patients’ private information.^42^ However, they also argue that where there is a pre-existing professional relationship, as between a clinician and their patient, equitable principles under the duty of confidence should take precedence, in particular the test of conscionability.^43^

In a 2019 article, Taylor and Wilson take issue with Linklaters’ conclusion, arguing that continued observance of equitable principles disregards the incorporation of Article 8 ECHR into English confidentiality law.^44^ They advocate for ‘reasonable expectations of privacy’ as the central test for a breach of confidence. In their view, the ‘reasonable expectations of privacy’ test offers a more sustainable and authentic approach to patient confidentiality, which could be reinforced with empirical deliberation into patients’ actual expectations around their health data.^45^ Dove, however, still sees the appropriate foundation of medical confidentiality as lying within equity.^46^

I agree with Taylor and Wilson’s criticisms of a medical confidentiality test based on the consciences of reasonable clinicians, particularly in their challenge to its continuing relevance. Article 8 ECHR centres the individual whose privacy is at stake, and not the conscience of the public authority employee who might interfere with it. To take the logic to its extremity, if ECHR rights were disapplied whenever individuals’ consciences were at ease, these rights would be of little constitutional utility in the Big Data landscape of the twenty-first century. The authors quote a convincing passage from Sedley LJ on the ‘structured approach’ to the proportionality of disclosure offered by Article 8, which he preferred to the conscience test.^47^

Where I differ from these authors, however, is that I do not believe that ‘reasonable expectations of privacy’ has, on the evidence of the two cases explored in this article, functioned well to protect patients’ information in a clear and accessible way. Admittedly, these authors are not alone in championing ‘reasonable expectations of privacy’ as a potential conduit for social responsiveness—Moreham also suggests the concept grounds the common law of privacy in societal values.^48^ Thus far, however, the impact of the test seems to bear out the concern expressed by Aidinlis—that the centrality of reasonable expectations in the UK privacy conception can have a restrictive effect on the scope of fundamental rights.^49^

Whether or not the correct test for confidentiality still derives from equitable principles, it is still clear that the scope of medical confidentiality is underpinned by Article 8 ECHR.^50^ Even if the Court of Appeal should have applied a different test to that of ‘reasonable expectations’ when determining confidentiality in *R (W, X, Y & Z)*,^51^ Article 8 will still be a foundational right that shapes how a different test (such as the tripartite test from *Coco v AN Clark*^52^) should be applied. As a domestic manifestation of the ECHR right to private and family life, medical confidentiality should reflect the proper scope of Article 8. This right should apply to the overwhelming majority of identifiable information the healthcare service holds about its patients, without nebulous loopholes deriving from broad ideas of ‘reasonableness’.

The next section uses the case of *R (W, X, Y & Z)* as a detailed illustration of how debating reasonableness creates gaps in legal protection, and why I argue for reasonable expectations of privacy to be presumed in medical records.

## III. CONFIDENTIALITY IN *R (W, X, Y & Z)*

In 2011, a change in Immigration Rules introduced a sanction for non-citizen NHS debtors.^53^ The NHS shared the data of thousands of patients with the Home Office, with the possibility of triggering detention at the UK border.^54^ After years of litigation,^55^ parliamentary criticism^56^ and public consultation^57^ around these data-sharing arrangements, they were eventually abandoned in 2018, with the Home Office agreeing to focus its monitoring on criminal offenders,^58^ rather than patients who had been unable to pay for their healthcare.

Even this brief history should help explain why it is concerning that the Court of Appeal ruled in *R (W, X, Y & Z) v Secretary of State for Health* that the claimant patients’ information was not private or confidential when passed from the NHS to the Home Office (via the Secretary of State). This aspect of the judgment is contentious and, I will argue, wrong.

The key part of Briggs LJ’s judgment comes at paragraphs [44–45]:

We do not see how overseas visitors who, before they are treated in an NHS hospital, are made aware of the fact that, if they incur charges in excess of £1,000 and do not pay them within 3 months, the Information may be passed to the Secretary of State for onward transmission to the Home Office for the stated immigration purpose can have any, still less any reasonable, expectation that the Information will not be transmitted in precisely that way … .

We therefore consider that the judge was right to hold that the Information is generally not private information vis-à vis the Secretary of State and the Home Office.

As an initial clarification: the above extract from the judgment uses the word ‘private’, seemingly because it was earlier held that ‘reasonable expectations of privacy’ was the relevant test for both privacy and confidentiality.^59^ The terms ‘private’ and ‘confidential’ are then used in a mostly interchangeable manner throughout the judgment. I agree with Dove, that the two terms are effectively conflated here.^60^ Had the Court used a test other than that of ‘reasonable expectations’, they *might* have considered more expansive accounts of confidentiality (see the discussion of *Egdell* in subsection A, below). The Court acknowledged the plethora of guidance to the effect that all identifiable information held by the NHS providers is confidential,^61^ but nonetheless disapplied privacy and confidentiality on the basis that disclosure should have been ‘reasonably’ anticipated by the patients.

Under the presumption advocated in this article, the identifiable information of W, X, Y and Z should have been considered private, with only clear evidence of irrelevance to/non-impact on their private lives displacing this presumption. This would require more than a dismissal of them as irrational in accessing healthcare they could not afford, because they may (or may not^62^) have been told about the consequences of unpaid NHS debt. The Court of Appeal’s ruling was not only flawed in its excessive emphasis on patients’ anticipation, to the detriment of considering the proper scope of private life.^63^ It also helped prolong the life of the NHS-Home Office information sharing, with the Government Ministers repeating the same logic in their response to the Health Committee’s concerns:

It is also important to consider the expectations of anybody using the NHS–a state-provided national resource. We do not consider that a person using the NHS can have a reasonable expectation when using this taxpayer funded service that their non-medical data, which lies at the lower end of the privacy spectrum, will not be shared securely between other officers within government in exercise of their lawful powers in cases such as these.^64^

This echo from the Government illustrates the implications of the Court of Appeal’s 2015 judgment for the thousands of patients who had their data shared with the Home Office between 2011 and 2018. Reasonableness is a broad, malleable concept which can all too easily be used to justify a much broader swathe of policies than that of proportionality, under Article 8(2) ECHR. It is the latter safeguard that patients deserve when their NHS data are used for purposes beyond their care, not a debate about their circumstantial reasonableness.

### A. The Article 8—confidentiality paradox

Had the Court of Appeal used a test other than that of ‘reasonable expectations’ in ruling on privacy and confidentiality, they might well have come to another conclusion. Hence the paradox that gives its subsection its name: the influence of Article 8 ECHR in English law has driven the adoption of a ‘reasonable expectations’ test which has also, paradoxically, made Article 8 easier to disapply. Its impact can be seen from the facts of the judgment.

Named only as W, X, Y and Z in the judgment, the claimants were all non-citizen ‘overseas visitors’ to the UK who had incurred NHS debts exceeding £1,000. They challenged the transmission of their personal information from the NHS to the Secretary of State for Health and Social Care, and then onwards to the Home Office. These were not ad hoc, exceptional disclosures but a routine channel of transmission via the Secretary of State, whereby overseas visitors with unpaid NHS debts could be liable to immigration sanctions.^65^ W, X, Y & Z’s application did not involve a claim for breach of confidence, but for judicial review of the State’s use of confidential patient information.

The High Court judgment explains that the NHS disclosures were set out in spreadsheets and contained the following categories of information^66^:

- Name
- Date of Birth
- Address
- Nationality
- Travel Document Number
- Amount of Debt
- Date of Debt
- NHS Body to which the Debt was owed

The information in question clearly identified the patients in question. To return to the statutory definition, identifiable patient information is confidential under section 251 NHS Act if that information was obtained or generated by a person who, in the circumstances, owed an obligation of confidence to that individual.^67^

This statutory definition does not specify the area of law under which this ‘person’ should owe a duty of confidence. It is not even explicit whether the obligation in question must be legal, as opposed to one of officially codified professional ethics. As explored in Section II, there is a breadth of law (contract, tort, equity etc) and professional guidance, which could each be a source of an ‘obligation of confidence’. As such, it seems overwhelmingly likely that the clinicians who generated W, X, Y & Z’s data owed them *some* species of confidence. This seems to have been the view of the British Medical Association (BMA), who were joined as an interested party in the Court of Appeal hearing of *R (W, X, & Z)*.^68^ The BMA were concerned with the High Court’s construction of the medical duty of confidence in ruling that the sharing of patient data with the Home Office was lawful. The organization has since been credited as instrumental in bringing an end to the routine NHS sharing of patient information with immigration authorities.^69^

The BMA’s guidance is consistent with the long-established precedent set in *W v Egdell*,^70^ in which a patient’s information was deemed confidential but its disclosure to the Home Office was justified on the basis that he had previously killed four people, and posed a future risk if released from psychiatric detention. The Court of Appeal’s 2015 ruling that W, X, Y & Z’s details were not confidential when transferred to the Home Office seems strikingly retrograde in comparison. In the 1989 case of *Egdell*, information relating to ‘W’ *was* considered confidential, albeit justifiably disclosed.^71^ This raises the counterintuitive possibility that the influence of Article 8 ECHR since 1998 has restricted the scope of medical confidentiality in the UK, by limiting it to judicial ideas of patients’ ‘reasonableness’ that would fall better within enquiries into the justification for disclosure.^72^ The contrast is particularly stark when the facts of *Egdell* are taken into consideration. In 1989, ‘W’ was a psychiatric inpatient who had killed four people, and expressed an ongoing interest in bomb-making. His risk of criminality was not necessarily a ‘health-related’ insight, but Bingham LJ still considered the disclosed information to be confidential, requiring justification under Article 8(2) for its disclosure:

No reference was made in argument before us … to the European Convention of Human Rights, but I believe this decision to be in accordance with it. I would accept that Article 8(1) of the Convention may protect an individual against the disclosure of information protected by the duty of professional secrecy. But Article 8(2) envisages that circumstances may arise in which a public authority may legitimately interfere with exercise of that right in accordance with the law and where necessary in a democratic society in the interests of public safety or the prevention of crime.

In 2015, the patient claimants named W, X, Y & Z were accused of no risk to public safety, or wrongdoing other than incurring NHS debts for unpaid healthcare. It is therefore striking that their rights were understood so sceptically under the ‘reasonable expectations of privacy’ test. The implementation of the Human Rights Act 1998 was (presumably) not intended to undermine the default confidentiality of patients’ data, or the consequent need for careful scrutiny of disclosure outside the NHS. To further the comparison with the pyro-enthusiastic W in *Egdell*, it is worth giving further consideration to the case of Z, whose NHS data was not deemed confidential vis-à-vis the Home Office in 2015.

### B. Z and SXB: the unreasonableness of the reasonable test

The situation of Z, the final unnamed applicant in *R (W, X, Y & Z)*, provides a key example of how transmission of NHS data to the Home Office can impact someone’s private life, regardless of whether it reveals information about their health. The details of her case, to the extent that they are narrated, can be found at paragraph 11 of the High Court judgment:

Z had an entry clearance to visit the United Kingdom lawfully to see her husband, but she was refused leave to enter apparently on the basis that she was pregnant and she was detained. She was later released and had her child in December 2011, but she was liable to charging for the maternity treatment she received. Her NHS debt of £2,550 for that treatment was communicated to UKBA.^73^

Thus, having been legally cleared to visit her husband, Z was detained at the border because of her pregnancy. She then entered a regulatory regime in which emergency services and family planning were free to all, but maternity care during birth incurred a charge which could leave her, and her new baby, liable to further detention on the basis of NHS debt.^74^

To appreciate the potential impact of disclosure to the Home Office on Z, it is necessary to look beyond the judgment in *R (W, X, Y & Z).* In a more recent case,^75^ the UK Border Authority’s (UKBA) detention of NHS debtors with their young children was found to be unlawful. The Court found a failure of the public sector equality duty, with the NHS debtors being disproportionately female.^76^ This is the kind of issue that a more robust scrutiny of justification (for example, under Article 8(2) ECHR) could have unearthed.

In SXB’s case—*R(MXK, EH, HH, SXB, and ALK) v Secretary of State for the Home Department*—the judgment opens with painstaking detail from the claimants’ evidence, including the dates, durations and circumstances of their detention. At paragraph 10, it is recounted that SXB (who incurred debts from treatment for several miscarriages and a stillbirth) was stopped at the border and detained for six hours with a young baby, for whom she was told she should have brought food. The Court did not rule on whether a breach of Article 8 ECHR had occurred in these cases, but only because the challenge succeeded on other grounds. From the text of the judgment,^77^ it does not appear controversial that Article 8 at least was engaged, as a determination on justification under Article 8(2) was the unresolved issue.

Z’s case has clear parallels with those of the women seeking judicial review of the UKBA’s decisions, but no privacy interest in her NHS data was acknowledged beyond its potential to reveal information about her health. The High Court acknowledged that it was ‘obviously unlawful’ that the nature of her treatment was accidentally disclosed,^78^ but did not otherwise find that her information engaged her right to private and family life under Article 8 of the ECHR. It can be inferred that the Court considered Z to have a legally protected interest in her treatment-related information because it revealed an aspect of her private life. But it was not accepted that she had an Article 8 interest in the ‘non-clinical’ information which could, in the hands of the Home Office, cause her to be detained with, or separated from, her family. This would arguably be a more significant interference with her right to private and family life than the mere disclosure that she had given birth in a hospital. As such, it is a stark example of the technicality with which the English Courts can calculate medical confidentiality, and how far it has drifted from the core purpose of Article 8.

This is not to say that the disclosure to the Home Office could not have been justified on public interest grounds under Article 8(2) ECHR, but this justificatory analysis should be separate from the scope of medical confidentiality. In an alternate line of reasoning the Court of Appeal did find that disclosure to the Home Office was justified under Article 8(2) later in the judgment.^79^ But how the Court arrived at this conclusion is questionable, given their focus on the claimants’ reasonableness, leading to terse justification such as:

A patient liable to charges will reasonably expect that, in the event of default, steps will be taken to enforce payment.^80^

The vagueness of the word ‘steps’ is concerning. Of course, the Court of Appeal is unlikely to have meant ‘any steps, including the disproportionate and discriminatory’. But herein lies the issue with dismissing such cases on the basis of the scope of Article 8(1), as determined by reasonable expectations. Reasonableness thus takes on an undue level of prominence—specifically the *patient’s* reasonableness—without consideration of the broader context, or particular features of the patient’s case.

In practice, I suggest it is likely that the clinicians who generated W, X, Y & Z’s information would have owed some obligation of confidence—be it ethical, regulatory, statutory, contractual, equitable or tortious, or an overlapping combination of these obligations. This should have been enough for the data to be considered ‘confidential patient information’ for the purposes of section 251 of the NHS Act 2006, without enquiry into the reasonableness of the claimants’ expectations. But for the avoidance of doubt, and protracted enquiry of heterogeneous doctrine, it could simply be presumed that identifiable patient data are confidential, and attract a reasonable expectation of privacy outside healthcare uses.

It is striking that a patient with a history of multiple homicide was protected by Article 8(1) via medical confidentiality in 1989, but not W, X, Y and Z in 2015. This contrast suggests not only a flaw in the later judgment, but a wider degradation of the medical duty of confidence as it is constructed in law. But this degradation has not occurred in a vacuum. The test of reasonable expectations which was applied in *R (W, X, Y & Z)* has pervaded across English information law since then, acting as a filter for legitimate concerns about impact and proportionality. The next section therefore shows how the application of the test within the MOPI tort also has consequences for secondary uses of patients’ data.

## IV. MISUSE OF PRIVATE INFORMATION: *PRISMALL V GOOGLE*

This section focuses on the 2023 High Court judgment in *Prismall v Google.* The case is used to demonstrate how the new MOPI tort does not negate the need for a presumption of reasonable expectations of privacy in secondary uses of NHS data. In short, the MOPI tort does not necessarily fill any gaps within the legal construction of medical confidentiality, as it also prompts consideration of a claimant patient’s reasonableness.

This judgment has since been upheld by the Court of Appeal.^81^ The Court of Appeal’s ruling does not fundamentally alter anything argued within this section. However, it warrants a couple of caveats. Firstly, the appellate judgment highlights that the construction of ‘reasonable expectations’ here was not made in a vacuum, but rather in conjunction with the particularities of the ‘lowest common denominator’ test for class actions. The Court emphasized that many of the individuals affected by the disclosure would, indeed, have had a reasonable expectation of privacy,^82^ had they not been judged according to a hypothetical ‘lowest common denominator’ patient. They even cast doubt on the ‘attractiveness’ of Google’s suggestion that the data of 1.6 million patients were no longer private, because two patients had agreed to altruistically publicize their treatment in the media for the sake of public awareness.^83^

A wider debate on the merits of the ‘lowest common denominator’ test is outside the scope of this paper. However, the criticism of the ‘reasonable expectations’ test is still pertinent here. If privacy in medical records were presumed by default, without reference to a ‘reasonableness’ debate, a defendant would require at least some evidence to rebut this presumption,^84^ even with the considerable assistance of the ‘lowest common denominator’ test.

### A. *NHS Royal Free and Google UK Ltd*

In 2023, the High Court struck out a class action claim against Google UK Ltd based on their alleged misuse of private information. This ‘MOPI’ claim was brought on behalf of 1.6 million patients of the Royal Free NHS Foundation Trust. The English High Court dismissed the claim on the (now familiar) ground that the patients lacked a realistic prospect of establishing a reasonable expectation of privacy.^85^

The data of these 1.6 million patients was used in 2015-16 to develop an application for acute kidney injury (‘AKI’), which was known as ‘Streams’. The detail of this arrangement between the NHS Royal Free Trust and Google only became public knowledge through journalistic investigation. The Trust had already announced the development of an app to help manage acute kidney injury in February 2016, but had not detailed the volume and nature of the patient data involved.^86^ It was thus a significant development when Hal Hodson reported that the data involved in developing this AKI application:

will include information about people who are HIV-positive, for instance, as well as details of drug overdoses and abortions. The agreement also includes access to patient data from the last five years.^87^

In a subsequent study with Julia Powles, Hodson notes that Royal Free itself claimed that AKI affected about one in six patients,^88^ arguably rendering most of the data transferred unnecessary for the stated purpose. Although Google stated that there was no separate dataset exclusively for patients with kidney conditions,^89^ an important question is whether the relevant data could have been extracted at source before being transferred to Google. Or, indeed, whether a transfer to Google’s servers was even necessary, if remote access or federated analysis were potential alternatives?

These are the kinds of question which should be posed in what is now termed a Data Protection Impact Assessment (DPIA),^90^ and was at the time (under the Data Protection Act 1998) called a Privacy Impact Assessment (PIA). Despite the change in terminology, the purpose of the assessment is the same: to evaluate a planned processing of special categories of personal data, identify risks to data subjects’ rights and freedoms, and establish whether the risk can be justified or mitigated.^91^ In this sense, a ‘DPIA’ (or ‘PIA’, in the old terminology) closely mirrors the questions of justification and proportionality prompted by Article 8(2) ECHR. It is obviously essential that the impact of processing is evaluated ahead of time, to identify risks before avoidable harm is suffered. This is a key reason why I argue for a legal presumption that Article 8 will apply to the secondary uses of patient data, so that proper evaluation and justification is also conducted by default. From the reporting, this does not appear to have occurred in advance of Royal Free’s disclosure to Google UK.

According to Powles and Hodson, a PIA was commenced on 8 October 2015 (ie about a week *after* the contract to share data was signed).^92^ A report from a third-party audit conducted by Linklaters LLP is silent as to when this assessment began, but notes that it was completed in January 2016,^93^ and that it was ‘relatively thin’ given the scale of the project. It seems fair to infer that the parties assumed that the disclosure of data was lawful, necessary and proportionate to their aims, as they only completed a formal evaluation of its impact after patient data was transferred.^94^

### B. The ICO’s approach to reasonableness

Before examining the ruling in *Prismall*, it is worth reviewing a related enforcement action brought by the UK’s Information Commissioner’s Office (‘ICO’), albeit against the relevant NHS Trust and not Google. The ICO’s published recommendations exemplify a more patient-centred application of the ‘reasonable expectations’ test, with ‘reasonableness’ construed more from the perspective of an ‘ordinary’ person. The Information Commissioner’s letter to the NHS Royal Free Trust^95^ outlines findings of multiple breaches of data protection law, and expresses concern at the lack of a full privacy impact assessment. If the NHS Royal Free Trust had conducted a prospective PIA (ie before contracting to share patient data), it is likely that an effective assessment would have captured issues of necessity and proportionality.

Many of data protection law’s requirements mirror the justificatory principles under Article 8(2): ie is the use necessary, lawful, and proportionate? Data protection law can be broader in scope than confidentiality and privacy laws, at least as it applies to digitally recorded information, as it covers all ‘personal data’ (ie information relating to an identified or identifiable individual).^96^ As we can see in the Royal Free case, the broader scope of data protection law made it more effective at capturing the flaws in the disclosure of patient data than the deployment of the MOPI tort in *Prismall.*

In short, the ICO was highly critical of Royal Free’s disclosure of patient data to Google. Their investigation was completed in 2017, with findings of multiple breaches of data protection law—including the principles of fairness and transparency.^97^ The letter sent publicly to the Royal Free NHS Trust does appear to apply a version of the ‘reasonable expectations’ test, albeit in a way which attempts to ground the concept in an envisaged perspective of an ‘ordinary’ representative patient:

For example, a patient presenting at accident and emergency within the last five years to receive treatment or a person who engages with radiology services and who has had little or no prior engagement with the Trust would not reasonably expect their data to be accessible to a third party for the testing of a new mobile application, however positive the aims of that application may be.^98^

Following the ICO’s findings, the Royal Free undertook to conduct a third-party audit of their ongoing transmission of patient data to Google UK. This was completed by Linklaters in 2018.^99^ The authors state that the nature of medical confidentiality in English law is unclear, given the multiple areas of law from which it could stem. They conclude that, in a medical context, the duty of confidentiality lies primarily in equity rather than tort, although they acknowledge a healthcare professional might be subject to both.^100^

This conclusion on the interrelation of tort and equity is not uncontroversial. Taylor and Wilson directly disputed Linklaters’ conclusion, citing caselaw to the effect that proportionality under the Human Rights Act 1998 is now a better touchstone than equitable principles of conscionability.^101^ Dove is more sympathetic to the continuing relevance of equity within confidentiality, but suggests this area of law is ambiguous and unsatisfactory.^102^ Unfortunately, in a sense, the litigation in *Prismall* was brought against Google UK Ltd, not the Royal Free NHS Trust, meaning the Court did not have an opportunity to consider the interrelation between the two legal obligations for healthcare providers. It is clear, however, that the logic applied within this class-action tort claim differed significantly from the ICO’s approach.

### C. The Prismall litigation

Given the outcome of the ICO’s investigation, the representative claimant in *Prismall* should have had a strong case in their class-action MOPI claim. Even though the ICO had explicitly stated that the disclosure to Google was contrary to patients’ reasonable expectations, the High Court found it inarguable that the 1.6 million affected patients shared a reasonable expectation of privacy.

Mrs Justice Williams, in fairness, had to navigate the ‘lowest common denominator’ test in her 2023 judgment. This emerges from the requirement under the Civil Procedure Rules for all claimants in a class action to share the ‘same interest’ in a claim.^103^ This effectively reduced this representative patient to a set of ‘variables’ which could be imputed across all 1.6 million people in the claimant class. These included 11 variables listed at paragraph 166 of the judgment, including:

- Attendance for a medical condition involving no ‘particular sensitivity or stigma’ (whatever this might mean);
- This non-sensitive disease nonetheless prompts an attempt to seek hospital treatment;
- No information about them is recorded after they speak to a receptionist, possibly because they leave without being treated;
- Their attendance in hospital is a matter of ‘public record’, possibly because they post about it on social media.

These hypothetical features undermine, in my view, the supposed ‘representativeness’ of this lowest common denominator patient. To identify a set of characteristics which *all* patients in the 1.6 million class would share, the court had to whittle down the imagined circumstances to a point which falls below the bar of a ‘normal’ patient experience.

The resulting hypothetical patient, who attends hospital for a trivial condition, leaves without being seen, and documents their full, uneventful day in A&E via social media, may be our hypothetical ‘lowest common denominator’ but they hardly represent the average patient. Even without the benefit of attendance statistics for the Royal Free NHS Trust, we can assume that the majority of patients attending hospital *do* see a clinician before they leave. And the majority do *not* post a full account of their treatment on social media.

The different conclusions reached by the High Court and the ICO can be attributed to their respective methods of constructing reasonable expectations of privacy. The Information Commissioner was at liberty to imagine the perspective of an ‘ordinary’ representative patient, whereas Mrs Justice Williams applied the precedent in *Lloyd v Google*^104^ to create an almost nonchalant ‘lowest common denominator’. Even outside of class actions, when a court does *not* have to construct reasonable expectations on a ‘lowest common denominator’ basis, the underlying concept of a reasonable perspective can still be contentious and exclusive. This is considered in the next subsection.

### D*. The reasonable man in English law*

The above analysis is not only a critique of how the High Court constructed ‘reasonable expectations of privacy’ in *Prismall.* It forms part of a broader argument, that this issue should not have been deliberated in the first place. If the Court had presumed that secondary uses of identifiable patient data attract a reasonable expectation of privacy, patients could have avoided discussions of reasonableness which are obscured by the long shadow of the ‘reasonable man’ in English common law.

Even outside the quirks of the ‘lowest common denominator’ test, therefore, an appeal to the concept of a ‘reasonable expectation’ can make it all too easy to summarily dismiss patients’ privacy rights. Before the class action in *Prismall* was issued, Linklaters LLP prepared an audit report for the Royal Free NHS Foundation Trust, as part of their response to the Information Commissioner’s criticisms.^105^ Within this report, the authors take a strikingly different approach to reasonable expectations compared to the ICO.

Linklaters also acknowledge Taylor and Wilson’s favoured test—the reasonable expectations of the patient—as an alternative (or supplementary) benchmark under the ‘MOPI’ tort. But they suggest that a patient ‘would reasonably expect the hospital to properly test the system before deploying it’.^106^ In a footnote, they add:

For example, if one were to ask the reasonable patient on the Clapham omnibus if they would expect a hospital to properly test its information technology systems before putting them into use, the patient might well testily suppress the question with an 'Oh, of course!’

The phrasing of this question to the fictitious bus passengers is strikingly euphemistic. There is ‘proper testing’ and there is ‘disclosure of HIV status to develop an unrelated kidney injury app’.^107^ But whatever the phrasing of this imaginary dialogue, its utility in determining the scope of patients’ privacy rights is questionable. For readers unfamiliar with the significance of this Clapham omnibus, the ‘man on the Clapham omnibus’ has been a figurative stand-in for an ordinary, reasonable perspective since 1903.^108^ In 1969, Megarry J famously decided that this reasonable man should ‘labour in equity’, to serve as a benchmark for the law of confidence: ‘the hard-worked creature, the reasonable man, may be pressed into service once more for I do not see why he should not labour in equity as well as at law’.^109^

This reasonable, ordinary man is indeed a hard-working, and obligingly malleable, figure of the judicial imagination. The shadow he has cast over English politico-legal culture stretches far beyond the Edwardian era. He is a figure of nostalgic affection for many English lawyers, but is nonetheless a highly subjective creature, whose imagined opinions offer only meagre defence (if any) against arbitrary interference with fundamental rights. In March 2019, for example, the *Financial Times* reported the bewilderment of EU officials at the UK Attorney General’s suggestion that the question of the Northern Irish (Brexit) backstop could be arbitrated according to the perspective of ‘an imaginary south London commuter’—a suggestion they ‘flatly rejected’.^110^ If the ultimate purpose of Article 8 of the ECHR is to prevent arbitrary interference with private life,^111^ such a highly subjective figure does not offer a reliable safeguard against arbitrariness. This presents an issue for those who have argued that ‘reasonable expectations’ can ground privacy law in contemporary social norms and values^112^; as valuable as it may be for judges to cite empirical evidence of public attitudes when they consider Article 8, they are by no means obliged (or even encouraged) to do so. In the USA, the ‘time-honored’ concept reasonable persons has also been criticized as an inadequate benchmark for healthcare regulation; suggesting the nebulousness of the concept is not unique to the English construction.^113^

The subjective, non-empirical nature of the ‘reasonable man’ is not a novel observation. It has been openly acknowledged and discussed by the English judiciary. As Lord Reed observed in *Healthcare at Home v Common Services Agency*:

It follows from the nature of the reasonable man, as a means of describing a standard applied by the court, that it would be misconceived for a party to seek to lead evidence from actual passengers on the Clapham omnibus as to how they would have acted in a given situation or what they would have foreseen, in order to establish how the reasonable man would have acted or what he would have foreseen. Even if the party offered to prove that his witnesses were reasonable men, the evidence would be beside the point. The behaviour of the reasonable man is not established by the evidence of witnesses, but by the application of a legal standard by the court.^114^

This judicial warning against canvassing fictitious bus passengers, under the guise of objective, empirical enquiry is well-founded. There can be a tempting malleability to these imaginary legal friends, which undermines the apparent objectivity of any ‘reasonableness’ they bring to English law.

This sensible, imaginary user of public transport who arbitrates all matters of social ambiguity can be seen as a composite figure, drawn from abstract ideas of fairness and the decision-maker’s own views, and thus fluid according to what ‘justice’ requires him to be. As Lord Radcliffe stated in *Davis Contractors Ltd v Fareham Urban District Council*:

The spokesman of the fair and reasonable man, who represents after all no more than the anthropomorphic conception of justice, is and must be the court itself.^115^

As the anthropomorphic conception of justice, the ‘reasonable man’ is formed by judges mentally conjuring up this person, and in the process of achieving some communion between his imagined perspective and what must surely be their own. The subtle blend of imagined ordinary perspective and the judge’s own view has been acknowledged by Lord Mance, speaking extra-judicially:

certainty cannot be restored to its perch by the lawyers’ habit of invoking apparently independent criteria—as when we say that we judge conduct by the standards of the man on the Clapham omnibus (or Oxford tube) or the reasonable bystander … . In the former case, the judge’s own instincts are likely to have a role.^116^

There may still be a useful role for the ‘reasonable man’ in English common law—and, clearly, this is a question beyond the scope of this article. The commentary cited above shows that the figure can be deployed with judicial self-awareness of its partiality. But here, in the context of secondary uses of health data, it is legitimate to expect a greater degree of certainty than the concept of reasonableness can deliver. Otherwise, patient privacy remains at the mercy of a very broad, scope-setting concept.

My argument for a rebuttable legal presumption of reasonable expectations of privacy, within secondary uses of identifiable patient data, is not made in a vacuum. By ending judicial deliberations of reasonableness, we would not only bring confidentiality and MOPI into closer alignment with the scope of data protection law (per *Prismall*), and the BMA’s view of medical confidentiality (per *W, X, Y & Z’s* case). It would also bring the English law’s approach to health data more closely in line with the approach of the ECtHR to Article 8 ECHR. This is explained in the final subsection, below.

### E. Reasonable expectations under Article 8 ECHR

The right to privacy emphasized here is set out in Article 8 ECHR. Article 8 falls into two parts: a right to privacy and a prohibition on public authorities’ interference with this right without due justification:

1. Everyone has the right to respect for his private and family life, his home and his correspondence.
2. There shall be no interference by a public authority with the exercise of this right except such as is in accordance with the law and is necessary in a democratic society … .

The ECtHR has characterized the central aim of Article 8 ECHR broadly, including the assurance that any state interference with privacy is not arbitrary. To prevent such arbitrary action, public authority use of citizens’ information should be subject to a structured inquiry of proportionality, including whether the interference is limited to the minimum necessary for a legitimate aim. The question of whether citizens have a ‘reasonable expectation of privacy’ has not featured as significantly in ECtHR judgments as it has in the UK.

One of the first reported references to ‘reasonable expectations of privacy’ in ECtHR jurisprudence comes in *Halford v United Kingdom*,^117^ being introduced via the submissions of the UK government. In this case, the government’s assertion that the claimant lacked a reasonable expectation of privacy, and that Article 8(1) should therefore not apply to the interception of her telephone calls, was unsuccessful.^118^

*Halford v UK* was not a unique outcome for the UK government. In *Peck v United Kingdom*^119^ and *Perry v United Kingdom*^120^ the ECtHR also rejected the UK’s submission that Article 8 was not engaged, because the claimant lacked a reasonable expectation of privacy. Similarly, in *Pay v United Kingdom*,^121^ the ECtHR decided to ‘proceed on the assumption, without finally deciding, that Article 8 [was] applicable’, casting some doubt on the UK Government’s assertion that the applicant lacked a reasonable expectation of privacy.^122^ The presence of a ‘legitimate expectation of privacy’ was debated in *Von Hannover v Germany*,^123^ but this judgment (i) concerned paparazzi photography, not state intrusion, and (ii) in any event, *did* hold that Article 8 was applicable. The *Von Hannover* judgment appears to be the genesis of the split between the ECtHR and the English courts on how to construct ‘reasonable expectations of privacy,’ as (unlike in *Campbell*) the Strasbourg court had no issue with finding a legitimate expectation of privacy in respect of ‘mundane’ activity, such as walking down the street.^124^

A helpful illustration of the ECtHR’s approach to secondary uses of health data comes from *Julien v France*.^125^ The ECtHR held that the central collection and retention of health records engaged Article 8. In this case, a record of admissions to psychiatric hospitals was retained by central government, who assured the Court that the records did not inform any measures taken against the patient but were retained for their benefit.

While the ECtHR agreed that the retention of psychiatric records was justified as a lawful and proportionate response to a legitimate aim, they still held that Article 8 was engaged. This sets an important precedent: the use of private information by the state does not need to be malign to engage the right to privacy, and it does not need to involve the security services or secret police. The precedent in *Julien v France* is consistent with subsequent cases in which Article 8 has been deemed applicable once a systematic or permanent record of personal information is created by a public authority.^126^ This is particularly true when this information could be exploited for multiple purposes, and so represents a multi-faceted risk to the data subject’s interests. Thus, in *S and Marper v United Kingdom* the mere storage of genetic and biometric data by the police was deemed to engage Article 8, due to the range of uses for which it could conceivably be used.^127^

The above review of Strasbourg authority illustrates why I characterize the ECtHR’s interpretation of Article 8 as more expansive, compared to the English emphasis on ‘reasonable’ claimants. Where patients’ information is systematically retained and re-used by the NHS, the duties of the health service as a collection of public authorities should apply by default. As such, the ECtHR’s recognition of the importance of medical information for Article 8 provides a more reliable standard for the application of the right—rather than scrutinizing the reasonableness of each individual patient’s expectations of privacy, based on their circumstances, or a contentious stock figure.

## V. CONCLUSION

To return to Lord Kerr’s words in *Re JR 38*, which were cited at the beginning of this article*:*

Von Hannover v Germany (2004) 40 EHRR 1 is not authority for giving reasonable expectation of privacy … unique status. It is true that … the court referred to the reasonable expectation of privacy but this was for the purpose of making clear that where there was such an expectation, that was a factor in favour of the engagement of article 8. The court did not suggest that, if there was no reasonable expectation of privacy, that would be determinative of the issue.^128^

Despite this warning, reasonable expectations of privacy have taken on an unhelpfully central position within the English conception of privacy rights. This article has explored the consequences of this gradually ossified test, particularly within attempts to litigate the secondary uses of patient data. I have argued that deliberation of patients’ ‘reasonableness’ has restricted their information rights well below the standards set by data protection law, the British Medical Association, and the European Court of Human Rights. I have therefore advocated for a rebuttable (evidential) legal presumption of reasonable expectations of privacy in secondary uses of patients’ data.

Article 8 should protect citizens from arbitrary interference in their private lives, and the most marginalized members of society are likely to need this protection the most.^129^ For this reason, I have argued for a legal presumption of reasonable expectations of privacy per Article 8(1) in the secondary uses of identifiable patient information. The complex, heterogeneous mass of vulnerabilities contained in this information should warrant the additional justificatory safeguard of Article 8(2), and the structured enquiry into proportionality this fundamental right entails.

## Footnotes

1
*Re JR 38’s Application for Judicial Review* [2015] UKSC 42; [2016] A.C. 1131, [56].

2
*Mr Andrew Prismall v Google UK Ltd* [2024] EWCA Civ 1516; 2024 WL 05077762, [62].

3 For example, see the usage within Regulation 2025/327 on the European Health Data Space, Chapter IV, arts 33–57.

4 ibid.

5
*R (on the application of W, X, Y and Z) v Secretary of State for Health and Secretary of State for the Home Department, and the British Medical Association* [2015] EWCA Civ 1034; [2014] 5 WLUK 528.

6
*Andrew Prismall v Google UK* [2023] EWHC 1169 (KB); [2024] 1 WLR 879.

7
*Andrew Prismall* (n 2).

8 ‘Public interest’ is used here as a shorthand for the grounds listed in art 8(2) ECHR, in particular the requirement that an interference with the right to private life be ‘necessary in a democratic society’.

9
*Campbell v MGN Ltd* [2004] UKHL 22; [2004] 2 AC 457 [21]. By ‘private life’, the Court was referring to the right to private and family life under art 8 ECHR.

10
*Bloomberg LP v ZXC* [2022] UKSC 5; [2022] AC 1158, [47].

11 ibid, [51].

12 If it is established that the right is engaged. *Sunday Times v The United Kingdom* (Application no. 6538/74) (1979).

13 Notably, in *S and Marper v The United Kingdom* (2008) 48 EHRR 1169, 4 December 2008, [70]–[86]. See also *Airey v Ireland* (1979) (1979–80) 2 EHRR 305; *Julien v France* (1991) App. No(s).14461/88, 9 July 1991.

14
*Glass v The United Kingdom* (2004) 39 EHRR 15.

15
*Bărbulescu v Romania |* [2017] 9 WLUK 42*; Antović and Mirković v Montenegro* [2017] 11 WLUK 675; *Karin Köpke v Germany* 53 EHRR SE26.

16 The earliest reference to ‘reasonable expectations of privacy’ (at least per the author’s review) being *Halford v The United Kingdom* (1997) EHRR 523. Followed by Se*e PG & JH v UK* (2008) 46 EHRR 51, [57], then *Copland v The United Kingdom* [2007] ECHR 253.

17
*Re JR 38* (n 1).

18
*Prismall* (n 2), [62].

19 In *Bloomberg* (n 10) the Supreme Court referred to a ‘legitimate starting point’ or ‘general rule’ [72].

20 In *Prismall* (n 2) the Court of Appeal noted that respecting medical confidentiality was a ‘vital principle’ under art 8 ECHR, [44].

21 In *Murray v Express Newspapers plc* [2008] EWCA Civ 446; [2009] Ch 481 the Court of Appeal disagreed with the ‘strong predisposition’ of the first instance judge against a person having a reasonable expectation of privacy while they walked down the street, [44].

22 Douglas Walton, *Burden of Proof, Presumption and Argumentation* (CUP 2014) 85.

23 Ian Dennis, *The Law of Evidence* (Sweet & Maxwell 2024) 12-024.

24 Raymond Emson, *Evidence* (Palgrave Macmillan 1999).

25 This was the ground for deviation from the ‘starting point’ given in *Prismall v Google* (n 2), [63].

26 The test for justification under art 8(2) ECHR.

27
*Bloomberg* (n 10), [67].

28
*Per Z v Finland* (1998) 25 EHRR 371, as cited in *Prismall* (n 2), [44].

29
*S and Marper v United Kingdom* (2008) 48 EHRR 1169, [66].

30
*Bloomberg* (n 10), [74].

31
*Murray* (n 21), [44].

32 To use the language of this article, not the court’s judgment.

33
*Weller & Ors v Associated Newspapers Ltd* [2015] EWCA Civ 1176; [2016] 1 WLR 1541, [63].

34 ES Dove, ‘Confidentiality, Public Interest, and the Human Right to Science: When can Confidential Information be Used for the Benefit of the Wider Community?’ (2023) 10 Journal of Law and the Biosciences 1, 8.

35 The terms ‘confidentiality’ and ‘confidence’ are used in a broadly interchangeable manner—in this paper and generally. Confidence may relate more to the duty itself, and confidentiality to the quality of the relevant information, but little rides on this distinction.

36 General Medical Council, ‘Confidentiality: good practice in handling patient information’ (January 2017) <https://www.gmc-uk.org/ethical-guidance/ethical-guidance-for-doctors/confidentiality> accessed 21 February 2025, para 9. Other healthcare professionals have similar professional ethics/regulatory standards, for example the Nursing & Midwifery Council’s ‘Professional standards of practice and behaviour for nurses, midwives and nursing associates’ (as updated 10 October 2018) <https://www.nmc.org.uk/globalassets/sitedocuments/nmc-publications/nmc-code.pdf> accessed 21 February 2025. For simplicity, registered healthcare workers are referred to collectively in this article as ‘clinicians’.

37 T Aplin and others, *Gurry on Breach of Confidence: The Protection of Confidential Information* (2nd edn, OUP 2012), [9.63].

38 N Richards and D Solove see the English breach of confidence action as a tort, not an equitable claim. See ‘Privacy’s Other Path: Recovering the Law of Confidentiality’ (2007) 96 Georgetown Law Journal 123, at 161. The authors of *Gurry* also suggest that confidentiality may stem from clinicians’ tortious duty of care, see n 12.

39 Despite Richards and Solove’s view (ibid), Megarry J refers to the ‘equitable doctrine of breach of confidential communication’ at [45] in his judgment in *Coco v AN Clark* [1969] RPC 41.

40 For example, the duty to have regard to the NHS Code of Confidentiality under the Health and Social Care Act 2012, s 263(6).

41 See *Z v Finland* (n 28).

42 Linklaters LLP, *Audit of the acute kidney injury detection system known as Streams* (17 May 2018, updated 7 June 2018) <https://www.royalfree.nhs.uk/news/royal-free-london-publishes-audit-streams-app**>** accessed 21 February 2025.

43 ibid 62.

44 MJ Taylor and J Wilson, ‘Reasonable Expectations of Privacy and Disclosure of Health Data’ (2019) 27 Medical Law Review 432.

45 ibid 433.

46 n 51, below.

47 Taylor and Wilson (n 44) 456.

48 NA Moreham, ‘Conversations with the Common Law: Exposure, Privacy and Societal Change’ (2021) 52 Victoria University of Wellington Law Review 3, 563–77, 576.

49 S Aidinlis, ‘The Right to be Forgotten as a Fundamental Right in the UK after Brexit’ (2020) 25 Communications Law 67.

50
*Z v Finland* (n 28).

51 As suggested by ES Dove, ‘Misuse of Private Information and the Common Law Right of Privacy: A New Frontier in Biomedicine?’ in Dove (ed), *Confidentiality, Privacy and Data Protection in Biomedicine* (Routledge 2024) 194–231, 207.

52 n 39, [47]. This test focuses on the (1) quality of confidence in information, (2) whether the circumstances of disclosure imparted a duty of confidence, and (3) whether disclosure was authorized. The importance of medical confidentiality under art 8 ECHR should be a key factor when applying limbs (1) and (2) of this test to patients’ data.

53
*R (on the application of W, X, Y and Z)* (n 5), [1].

54 Health and Social Care Committee, *Memorandum of understanding on data-sharing between NHS Digital and the Home Office* (HC 2017-19, 677).

55 n 70.

56 Letter from Dr Sarah Wollaston MP to Sarah Wilkinson (29 January 2018): <https://publications.parliament.uk/pa/cm201719/cmselect/cmhealth/Correspondence/Wilkinson-2018-01-29.pdf> accessed 17 October 2025.

57 Public Health England, ‘Data-sharing MoU between NHS Digital and Home Office: call for evidence’ (15 February 2018) <https://www.gov.uk/government/calls-for-evidence/data-sharing-mou-between-nhs-digital-and-home-office-call-for-evidence> accessed 21 February 2025.

58 Home Office, ‘Home Office in the Media: 10 May 2018’ (10 May 2018): <https://homeofficemedia.blog.gov.uk/2018/05/10/home-office-in-the-media-10-may-2018> accessed 21 February 2025.

59
*R (on the application of W, X, Y and Z)* (n 5), [26].

60 n 51.

61
*R (on the application of W, X, Y and Z)* (n 5), [39].

62 At [43] the Court accepted that forewarning was only deemed ‘best practice’ under the guidance, and not therefore essential.

63 I might reasonably anticipate an intrusion into my family home, for example, but this anticipation alone does not mean that my home ceases to be private. Timothy Pitt-Payne QC also calls this aspect of the judgment ‘surprising’, and questions whether (eg) publication of medical records on the internet would also lack a reasonable expectation of privacy, simply because the patient had been informed in advance: ‘Panopticon’ (16 October 2015) <https://panopticonblog.com/2015/10/16/privacy-patients-and-payments-information-sharing-in-the-court-of-appeal/> accessed 21 February 2025.

64 Health and Social Care Committee (n 54), [38].

65
*R (on the application of W, X, Y and Z)* (n 5) [2]–[4].

66
*R (on the application of W, X, Y & Z) v Secretary of State for Health* [2014] EWHC 1532 (Admin); (2014) WL 1220234, [35]–[36].

67 NHS Act 2006, s 251(11)(b).

68
*R (on the application of W, X, Y and Z)* (n 5) [10].

69 British Medical Association, Brief for Annual representatives meeting, *The ‘hostile environment’* (2021) <https://www.bma.org.uk/media/4561/bma-arm-briefing-hostile-environment-arm2021.pdf> accessed 21 February 2025, 2.

70 [1990] Ch 359.

71 ibid [417].

72 In this regard, I concur with Taylor and Wilson (n 44) 451.

73 n 67, [11].

74 See below, n 76.

75
*R (MXK, EH, HH, SXB, and ALK) v Secretary of State for the Home Department* [2023] EWHC 1272 (admin); (2023) WL 03694924.

76 ibid, [80]–[91].

77 ibid, [79].

78 n 67, [11].

79
*R (on the application of W, X, Y and Z)* (n 5), [51].

80 ibid, [43].

81
*Andrew Prismall* (n 2).

82 ibid, [8].

83 ibid, [64].

84 See above, Section II subsection B.

85 n 6, [168].

86 See Powles and Hodson (n 88) 352 below.

87 H Hodson, ‘Revealed: Google AI has Access to Huge Haul of NHS Patient Data’ (29 April 2016) *New Scientist*, at: <https://institutions.newscientist.com/article/2086454-revealed-google-ai-has-access-to-huge-haul-of-nhs-patient-data/> accessed 21 November 2024.

88 J Powles and H Hodson, ‘Google DeepMind and Healthcare in an Age of Algorithms’ (2017) 7 Health Technology 351.

89 Hodson (n 87).

90 Regulation (EU) 2016/679 of the European Parliament and of the Council of 27 April 2016 on the protection of natural persons with regard to the processing of personal data and on the free movement of such data (UK General Data Protection Regulation), art 35.

91 Information Commissioner’s Office, ‘Data protection impact assessments’, at: <https://ico.org.uk/for-organisations/uk-gdpr-guidance-and-resources/accountability-and-governance/guide-to-accountability-and-governance/data-protection-impact-assessments/> accessed 21 November 2024.

92 Powles and Hodson (n 88) 356.

93 Linklaters LLP (n 42) 30.

94 Reports differ, but the patient data appears to have been transferred October–November 2015, with a live feed established thereafter. Linklaters LLP (n 42) 15.

95 Elizabeth Denham, Letter to Sir David Sloman (3 July 2017) <https://ico.org.uk/media/2014353/undertaking-cover-letter-revised-04072017-to-first-person.pdf> accessed 21 February 2025, 6.

96 UK GDPR, art 4(1).

97 ibid.

98 ibid 5.

99 Linklaters LLP (n 42).

100 ibid 62.

101 Taylor and Wilson (n 44) 456.

102 n 51, 197.

103 CPR, 19(8)(1). See *Prismall* (n 6) [6].

104 [2021] UKSC 50; [2022] AC 1217.

105 Linklaters LLP (n 42).

106 ibid 63.

107 See Hodson (n 87).

108
*McQuire v Western Morning News* [1903] 2 K.B. 100 at 109.

109
*Glass* (n 14).

110 A Barker, ‘“Man on the Clapham omnibus” Rides into Brexit Talks’ *Financial Times* (7 March 2019) <https://www.ft.com/content/f10bcf8a-40d7-11e9-9bee-efab61506f44 (last visited 20 September 2024)> accessed 21 February 2025.

111 As discussed below in the context of the jurisprudence of the ECtHR.

112 See Taylor and Wilson (n 44), and Moreham n 48.

113 J Banja, ‘Reasonable Persons, Autonomous Persons, and Lady Hale: Determining a Standard for Risk Disclosure’ (2020) 50 The Hastings Center Report 2, 25–34.

114 [2014] UKSC 49; [2014] 4 All E.R. 210 [3].

115 [1956] AC 696 [728].

116 Lord Justice Mance, ‘Should the Law be Certain?’ Oxford Shrieval Lecture (11 October 2011) <https://supremecourt.uk/uploads/speech_111011_342362219c.pdf> accessed 21 February 2025.

117 n 16.

118 ibid [45].

119 (2003) 36 EHRR 41, [53]–]63].

120 (2004) 39 EHRR 3.

121 (2009) 48 EHRR SE2 [12].

122 ibid [10].

123
*Von Hannover v Germany* (2005) 40 EHHR 1.

124 K Macmillan, ‘Baby Steps’ (2008) 13 Communications Law 3, 72–75.

125 n 13.

126 See *PG & JH v UK* (2001) App No 44787/98, 25 September 2001 [57].

127 (2008) 48 EHRR 1169, 4 December 2008 [70]–[86].

128
*Re JR* (n 1) [57].

129 G Laurie, ‘Is Privacy Egregiously Wrong? Reflections on a Concept that can Make or Break Constitutions’ in Dove (ed), (n 51) 37.

